# Algorithms for sequential interpretation of a malaria rapid diagnostic test detecting two different targets of *Plasmodium* species to improve diagnostic accuracy in a rural setting (Nanoro, Burkina Faso)

**DOI:** 10.1371/journal.pone.0211801

**Published:** 2019-02-13

**Authors:** Francois Kiemde, Massa dit Achille Bonko, Marc Christian Tahita, Petra F. Mens, Halidou Tinto, Henk D. F. H. Schallig, Michael Boele van Hensbroek

**Affiliations:** 1 Institut de Recherche en Science de la Santé-Unité de Recherche Clinique de Nanoro, Nanoro, Burkina Faso; 2 Amsterdam University Medical Centers, Academic Medical Centre, University of Amsterdam, Department of Medical Microbiology, Parasitology Unit, Amsterdam, The Netherlands; 3 Global Child Health Group, Amsterdam University Medical Centers, Academic Medical Centre, University of Amsterdam, Amsterdam, The Netherlands; Quensland University of Technology, AUSTRALIA

## Abstract

**Background:**

Malaria rapid diagnostic tests (RDT) have limitations due to the persistence of histidine-rich protein 2 (HRP2) antigen after treatment and low sensitivity of *Plasmodium* lactate dehydrogenase (*p*LDH) based RDTs. To improve the diagnosis of malaria in febrile children, two diagnostic algorithms, based on sequential interpretation of a malaria rapid diagnostic test detecting two different targets of *Plasmodium* species and followed by expert microscopy, were evaluated.

**Methods:**

Two diagnostic algorithms were evaluated using 407 blood samples collected between April and October 2016 from febrile children and the diagnostic accuracy of both algorithms was determined. Algorithm 1: The result of line T1-HRP2 were read first; if negative, malaria infection was considered to be absent. If positive, confirmation was done with the line T2-*p*LDH. If T2-*p*LDH test was negative, the malaria diagnosis was considered as “inconclusive” and microscopy was performed; Algorithm 2: The result of line T2-*p*LDH were read first; if positive, malaria infection was considered to be present. If negative, confirmation was done with the line T1-HRP2. If T1-HRP2 was positive the malaria diagnosis was considered as “inconclusive” and microscopy was performed. In absence of malaria microscopy, a malaria infection was ruled out in children with an inconclusive diagnostic test result when previous antimalarial treatment was reported.

**Results:**

For single interpretation, the sensitivity of *Pf*HRP2 was 98.4% and the specificity was 74.2%, and for the *p*LDH test the sensitivity was 89.3% and the specificity was 98.8%. Malaria was accurately diagnosed using both algorithms in 84.5% children. The algorithms with the two-line malaria RDT classified the test results into two groups: conclusive and inconclusive results. The diagnostic accuracy for conclusive results was 98.3% using diagnostic algorithm 1 and 98.6% using algorithm 2. The sensitivity and specificity for the conclusive results were 98.2% and 98.4% for algorithm 1, and 98.6% and 98.4% for algorithm 2, respectively. There were 63 (15.5%) children who had an “inconclusive” result for whom expert microscopy was needed. In children with inconclusive results (*Pf*HRP2+/*p*LDH- only) previous antimalarial treatment was reported in 16 children with malaria negative microscopy (16/40; 40%) and 1 child with malaria positive microscopy (1/23; 4.3%).

**Conclusion:**

The strategy of sequential interpretation of two-line malaria RDT can improve the diagnosis of malaria. However, some cases will still require confirmative testing with microscopy or additional investigations on previous antimalarial treatment.

## Background

Nowadays, malaria parasite specific antigen-detecting rapid diagnostic tests (RDTs) are considered essential tools in routine practice for the management of febrile illness in malaria endemic settings [1] [2] [3]. The short time needed to obtain a diagnostic result (within 30 minutes), the simple read-out system and easy performance (no need for a conventional laboratory or extensive training), combined with relative low costs have contributed to the acceptance of this method in differentiating malaria fever from other causes of fever. The most commonly used RDTs in Africa, including Burkina Faso, are the *Plasmodium falciparum* histidine-rich protein 2 (*Pf*HRP2) based RDT, which is specific for *Plasmodium falciparum* only, and the *Plasmodium* lactate dehydrogenase (*p*LDH) detecting RDT, which can detect 4 human *Plasmodium* species (*Plasmodium falciparum*, *P*. *ovale*, *P*. *malariae* and *P*. *vivax*) or a combination test, which has two test lines (a *P*. *falciparum*-specific line and an all human *Plasmodium*-test line).

In malaria endemic settings, several studies have been conducted to assess the sensitivity and specificity of *Pf*HRP2 and *p*LDH-based RDTs using microscopy as the gold standard [4] [5] [6] [7]. Most of these studies reported a relative low specificity for *Pf*HRP2 detecting RDTs and low sensitivity for *p*LDH detecting tests (<95%). Consequently, *Pf*HRP2 is useful for active detection of new malaria infection but unsuitable for monitoring parasite clearance following antimalarial treatment due to the persistence of HRP2 antigen in the blood for up to four weeks and over, which is causing false positive *Pf*HRP2 results and information on prior drug use should always be collected [4] [5] [7] [8] [9] [10] [11]. On the other hand, *p*LDH is metabolized within one week following antimalarial treatment and RDTs based on detection of this particular antigen were found to be suitable for monitoring parasite clearance, but less useful to detect new infections with a relatively low parasitaemia [12]. As a consequence, a malaria positive *Pf*HRP2 based RDT may not signal also for another cause of fever and a malaria negative *p*LDH based RDT may not detect a low malaria parasitemia. So, the incidence of false positive *Pf*HRP2 and false negative *p*LDH tests has created a diagnostic dilemma, often resulting in a loss of trust and non-adherence of health workers for malaria RDT results in general [13].

To circumvent this dilemma, sequential reading/interpretation of the two-line RDT detecting HRP2 and *p*LDH, specific of *Plasmodium* species, has been proposed.

## Methods

### Study site

The study was performed in the health district of Nanoro, central-west of Burkina Faso. Samples and data were collected in three peripheral health facilities (Nanoro, Nazoanga and Seguedin), and in the referral hospital, the Centre Médical avec Antenne Chirurgicale (CMA) Saint Camille of Nanoro. The only diagnostic tool available for the routine diagnosis of fever in patients seeking heath care in these clinics is a malaria RDT. The RDTs used in government clinics are one based on the detection of *Pf*HRP2 (manufacturers: SD Bioline: Standard Diagnostics, Hagal-Dong, Korea; CareStart: Access Bio, Inc.). The management of uncomplicated medical cases, including uncomplicated malaria (positive malaria RDT), is done at the health facilities by nurses according to the national guideline of integrated management of childhood illness [14]. Suspected malaria infection is the first reason for consultation in children under-5 years of age in this region and predominately occurs during the rainy season between July and November. The annual prevalence of malaria in children under-5 years of age has previously been determined to be 49.7% [15].

### Study design

This study was conducted in the framework of a large program aiming to improve the diagnosis and management of non-malaria fevers in children under-5 years of age [15]. Briefly, all febrile children (axillary temperature ≥37.5°C) under-5 years of age presenting in one of the three health facilities or the referral hospital between April and October 2016 were invited to participate in the study. Non-malaria infections which can be a probable cause of fever, such as bacterial bloodstream infection, gastroenteritis infection and urinary tract infection, were also investigated in the framework of this large survey [15]. Written informed consent was obtained from parent/guardian before any data or clinical specimen was collected. Basic demographic data of the included children and information on prior treatment, including antimalarials, in the two weeks before enrolment where collected.

For the purpose of the study a venous blood sample was collected in an Ethylene Diamine Tetra Acetic acid (EDTA) tube from each participant and specimens were transported to the Central Laboratory of the Clinical Research Unit of Nanoro (CRUN) for diagnostic (re-)testing (expert malaria microscopy and RDT’s to detect *Pf*HRP2 and *p*LDH). Malaria RDTs were ordered, stored and transported as recommended by the manufacturer (17–30°C). The patients were managed according to the national guideline and malaria RDT provided by the National Malaria Control Program (NMCP). The study was approved by the National Ethics Committee for Health Research of Burkina Faso (Deliberation N°2014-11-130).

### Laboratory procedure

#### Malaria rapid diagnostic test

A volume of 5 μl of a blood sample collected in an EDTA tube was used to perform the malaria RDT. A two-line malaria RDT detecting both *Pf*HRP2 and *p*LDH (SD Ag Bioline Malaria Ag *P*.*f/Pan*: Standard Diagnostics, Hagal-Dong, Korea; Lot number: 05EDC002A) with different lines specific for each target antigen and an internal control was used. The tests were performed and results recorded by a trained laboratory technician. A second trained technician also read and recorded the results of the RDT immediately after test execution and within the time limit set by the manufacturer. In case of discordance a third expert would be consulted to reach a decision. The test used in the present study went through the last WHO-FIND lot testing round 7 (Catalogue number: 05FK60) [16].

The performance of *Pf*HRP2 and *p*LDH were interpreted separately for the purpose of the study. In addition, the performance of the two-line malaria RDT was also calculated according to the manufacturer’s instructions for the interpretation of the three lines malaria RDT (the test is positive if the at least one line of T1-HRP1 and T2-*p*LDH is positive and negative in case of absence of any positive line for the different targets).

To assess the performance of the proposed diagnostic algorithms including *Pf*HRP2 and *p*LDH, the three lines of the employed malaria RDT were used. The test was valid if the internal control line was positive. The line T1-HRP2 is specific to *Plasmodium falciparum* and the line T2-*p*LDH is positive when *P*. *falciparum*, *P*. *malariae*, *P*. *ovale* or *P*. *vivax* is present. For the purpose of the study the results of the specific HRP2-line or the specific *p*LDH-line where separately recorded on a case report form (CRF). The following two diagnostic algorithms were considered within the recommended time of the manufacturer:

- Algorithm 1 and interpretation: Read line T1-HRP2 and confirmation with line T2-pLDH

**Fig 1:**
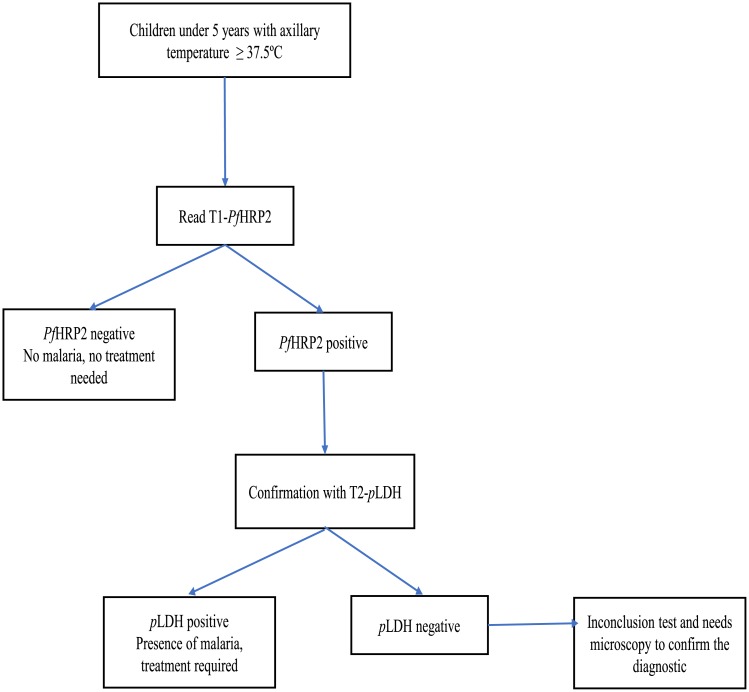
Proposed algorithm 1 for the diagnosis of malaria: Read T1-*Pf*HRP2 first and confirm the T1-*Pf*HRP2 positive line results with T2-*p*LDH line.

For the interpretation for algorithm 1, the results of the line T1-HRP2 of blood samples tested form febrile children were read firstly: The absence of positive line T1-HRP2 indicates the absence of malaria infection and a confirmation with the line T2-*p*LDH is not required. However, the presence of a positive line for *Pf*HRP2 indicates the necessity to confirm by reading the line T2-*p*LDH testing. The presence of positive line T2-*p*LDH (next to the positive line T1-HRP2) hence indicates the presence of malaria infection. The absence of a positive line T2-*p*LDH indicates that the diagnostic testing with RDTs was “inconclusive” and subsequently it was necessary to confirm the diagnosis with malaria expert microscopy (Fig 1).

- Algorithm 2 and interpretation: Read line T2-pLDH and confirmed with the line T1-PfHRP2

**Fig 2:**
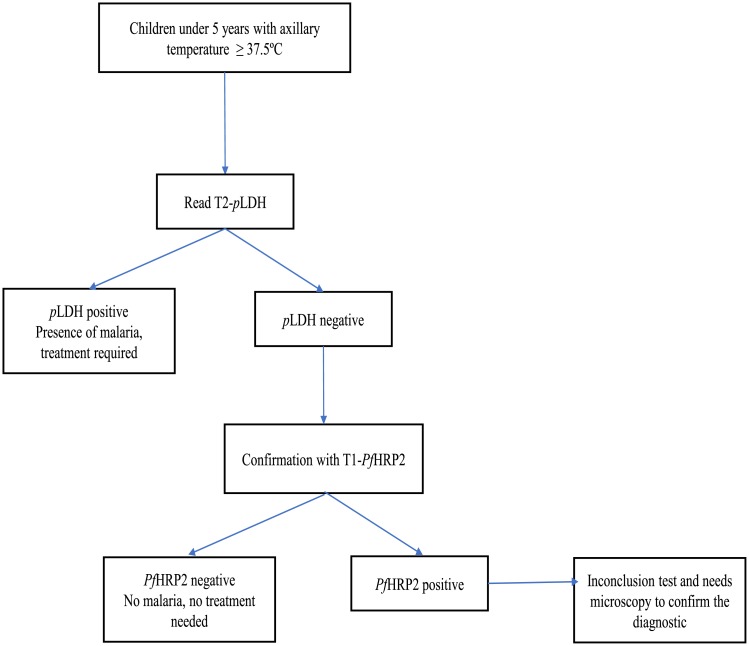
Proposed algorithm 2 for the diagnosis of malaria: Read T2-*p*LDH and confirm the T2-*p*LDH negative line results with T1-*Pf*HRP2 line.

For the interpretation of algorithm 2, the results of the line T2-*p*LDH of blood samples tested from febrile children were read firstly: The presence of a positive line T2-*p*LDH indicates the presence of malaria infection and a confirmation with the line T1-HRP2 is not required. However, the absence of positive line T2-*p*LDH indicates the necessity to confirm with the line T1-HRP2. The absence of positive line T1-HRP2 (next to the absence of positive line T2-*p*LDH) indicates the absence of a malaria infection. The presence of positive line for T1-HRP2 indicates that the diagnostic testing with RDTs was “inconclusive” and that it was necessary to confirm the diagnosis with malaria microscopy (Fig 2).

If malaria microscopy to confirm the diagnosis in children with inconclusive diagnostic results is not available, additional information on previous antimalarial treatment within the past 2 weeks was used to exclude a malaria infection.

#### Microscopy

Malaria diagnosis by microscopy was done by expert microscopists at CRUN who are subjected to regular external quality control (National Institute of Communicable Diseases/World Health Organization; NICD/WHO). At CRUN only accredited microscopists are authorized to read malaria slides [17]. Thick and thin blood smears were prepared (in duplicate) from blood collected in EDTA tubes as previously described. Thick blood smears were considered negative when the examination of 100 field per thick film did not reveal the presence of any asexual parasites. Each blood slide was read and parasite densities counted by two independent readers and in case of discrepancy (positive vs negative, difference in *Plasmodium* species, difference in parasite density >Log10 or ratio >2 in case of parasite density ≤400 or >400/μl, respectively) by a third reader [15]. These results were expressed as asexual parasites per microliter by using the patient’s white blood cells (WBC) count. Positive microscopy results were recorded as the geometric means of the two reader’s results or the geometric means of the third reading and the closest reading of the two other readings. Microscopists were blinded from the malaria RDT results. A selection of slides (5%) was re-read by an independent expert microscopist for quality assurance.

#### Full blood counts

Full blood cell counts were done by using blood samples collected in EDTA tubes and a Sysmex XS1000i (Sysmex Corporation, Kobe, Japan) according to manufacturer’s instructions.

#### Diagnosis of non-malaria infections

The methods applied for the diagnosis of non-malaria infections were in detail described previously [15]. Blood and urine specimen were systematically collected for bacterial culture, and stools for rotavirus and adenovirus tests.

### Data analysis

Data was entered in Excel 2016. Statistical analysis was performed by using R software version 3.3.1 (R Foundation for Statistical Computing, Vienna, Austria). For the purpose of this study, malaria microscopy was considered as gold standard. Sensitivity, specificity and predictive values were calculated for *Pf*HRP2, *p*PDH, the two-line malaria RDT (*Pf*/Pan) as recommended by the manufacturer and the two algorithms proposed. Proportion was used to describe qualitative data. For the quantitative data median was used to perform the description. Wilcoxon Rank Sum test was used to compare the median by estimating the p-value (p≤0.005) as statistically significant. Agreement between diagnostic approaches was determined by calculating Kappa (κ) values with 95% confidence intervals by using GraphPad software (https://www.graphpad.com/quickcalcs/).

## Results

### Characteristics of the study population

A total of 407 children under 5 years of age with documented fever were included in the study (Table 1). Of these children, 56.8% (231/407) were males and 25.6% (104/407) was under the age of 12 months. *Plasmodium falciparum* was found by expert microscopy in 60.0% (244/407) of the blood slides, and 5 *falciparum* patients were co-infected with other malaria species (four with *P*. *malariae* and one with *P*. *ovalae*). The median parasite density was 39,847 (Interquartile range [IQR]: 7,828–95,369).

**Table 1:** Characteristics of the study population (children under the age of 5 years with an axillary temperature of >37.5°C).

Characteristic	n (%)
**Gender**	
**Male**	231 (56.8)
**Female**	176 (43.2)
**Age (months)**	
≤**12**	104 (25.6)
>**12**	303 (74.4)
**Malaria prevalence (expert microscopy)**	244 (60.0)

Performance of two-line malaria RDT detecting PfHRP2/pLDH (P.f/Pan) compared to expert microscopy.

Expert microscopy was, for the purpose of this study, considered as the gold standard. Based on the manufacturer’s instructions, the *P*.*f/Pan*-based RDT was able to accurately diagnose 362 cases: i.e. 241 true positive cases (59.2%) and 121 true negative cases (29.7%). However, the *P*.*f/Pan*-based RDT had 42 (10.3%) false positive results and 3 (0.7%) false negative results (Table 2). The performance of *Pf*HRP2 and *p*LDH are also reported on Table 2.

**Table 2:** Diagnostic performance of *Pf*HRP2, *p*LDH and *Pf*/Pan compared with expert microscopy (gold standard) for the diagnosis of *Plasmodium falciparum* malaria.

	True positive	True negative	False positive	False negative
***Pf*HRP2**	240 (59.0)	121 (29.7)	42 (10.3)	4 (1.0)
***p*LDH**	218 (53.6)	161 (39.5)	2 (0.5)	26 (6.4)
***Pf*/Pan**	241 (59.2)	121 (29.7)	42 (10.3)	3 (0.7)

*P*.*f/Pan* = two-line malaria RDT detecting *Pf*HRP2 and *p*LDH; *Pf*HRP2 = *Plasmodium falciparum* specific histidine-rich protein 2; *p*LDH = *Plasmodium* lactate dehydrogenase.

The diagnostic accuracy of the *P*.*f/Pan*-based RDT results compared to expert microscopy as gold standard are presented in table 3. The sensitivity, specificity, positive predictive value and negative predictive value were 98.8% (241/244), 74.2% (121/163), 85.2% (241/283) and 97.6% (121/125) respectively. The agreement between *Pf*HRP2 testing and expert microscopy was considered to be “good; kappa value = 0.760 (95% CI 0.696 to 0.825; SE of kappa = 0.033). The sensitivity, specificity, positive predictive value and negative predictive value were respectively 98.4%, 74.2%, 85.1% and 96.8% for *Pf*HRP2 target and 89.3%, 98.8%, 99.1% and 86.1% for *p*LDH target (Table 3).

**Table 3:** Diagnostic accuracy of *Pf*HRP2, *p*LDH and *P*.*f/Pan* compared with expert microscopy (gold standard) for the diagnosis of *Plasmodium falciparum* malaria.

	Sensitivity	Specificity	PPV	NPN
***Pf*HRP2**	98.4 (95.9–99.5)	74.2(66.8–80.8)	85.1(81.5–88.1)	96.8(91.9–98.8)
***p*LDH**	89.3(84.8–92.9)	98.8(95.6–99.8)	99.1(96.5–99.8)	86.1(81.1–89.9)
***Pf*/Pan**	98.8(96.3–99.8)	74.2(67.0–80.4)	85.2(80.5–88.86)	97.6(92.8–99.5)

*P*.*f/Pan* = two-line malaria RDT detecting *Pf*HRP2 and *p*LDH; PPV = positive predictive value; NPV = negative predictive value.

### Diagnostic performance of algorithm 1 and 2

The diagnostic performance of algorithm 1 and 2 was comparable (Table 4). Algorithm 1 (read firstly the line T1-HRP2, followed by confirmation with the line T2-*p*LDH) resulted in 344 RDT confirmed diagnostic results: 219 positive cases and 125 negative cases. There were 63 “inconclusive” results (15.5% of all cases) that would require a final confirmation with microscopy if this algorithm would be applied. Algorithm 2 (read firstly the line T2-*p*LDH, confirmation with the line T1-HRP2) resulted also in 63 “inconclusive” results, and 220 positive and 124 negative cases. The algorithm 1 and 2 classified the diagnostic results in two groups: the conclusive diagnostic results in 84.5% (344/407) of children tested for which any additional information is not required to confirm malaria infection and the inconclusive diagnostic results in 15.5% (63/407) of children tested for which additional investigation is required following the algorithms. The conclusive results had higher median of parasites density of 45746 parasites/μl (IQR: 16618–101972) compared with inconclusive results with median of parasite density 778 parasites/μl (IQR: 119–1059.75) (p<0.0001).

**Table 4:** Summary of the diagnostic performance of algorithm 1 and 2.

	Screening		Confirmation		Results of the applied algorithm		
	Positive	Negative	Positive	Negative	Positive	Negative	Inconclusive**
**Algorithm 1**	282*	125	219	-	219	125	63
**Algorithm 2**	220	187*	-	124	220	124	63

*Needs to be confirmed by the second line of the two-line malaria RDT.

**Needs confirmation with malaria microscopy as the result of the two rounds of sequential read/interpretation with the two-line malaria RDTs is inconclusive.

There were thus 63 “inconclusive” test results with either employed diagnostic algorithm. For a final diagnostic result, these cases (15.5% of the whole study population) should be retested with expert microscopy. In the case of algorithm 1 and 2, these “inconclusive” test results comprised 23 cases that were found positive with expert microscopy and 40 cases that were reported as negative. Previous antimalarial treatments were collected within 2 weeks preceding inclusion. Despite the limit (HRP2 antigens can persist over 4 weeks), in children with inconclusive test (*Pf*HRP2+/*p*LDH- only), previous antimalarial treatments were reported in 16 children with malaria negative microscopy (16/40; 40%) and 1 child with malaria positive microscopy (1/23; 4.3%). Moreover, non-malaria infections (bacterial bloodstream infection, viral gastroenteritis infection and urinary tract infection) were diagnosed in 10 children with malaria negative microscopy (10/40; 25%) and 2 children with malaria positive microscopy (2/12; 8.7%; parasite density of 32 and 78 parasites/μl).

The agreement between expert microscopy and the two diagnostic algorithms on the 344 conclusive test results (i.e. those that were not requiring confirmation with microscopy) is summarized in Table 5. The frequency of true positives was similar in algorithm 1 and 2 (63.1% and 63.4% respectively) as well as the frequency of true negatives (35.2% for the both). Furthermore, the frequency of malaria false positives was similar in algorithm 1 and 2 (0.6%) as well as the frequency of false negatives (1.2% versus 0.9% respectively). The agreement between expert microscopy and diagnostic algorithm 1 (using the results of the “conclusive” RDT test results only) was “very good” (Kappa = 0.962; 95% CI 0.932 to 0.992; SE of kappa = 0.015). Furthermore, the agreement between diagnostic algorithm 2 and expert microscopy was in this case also “very good” (Kappa = 0.968; 95%CI 0.941 to 0.996; SE of kappa = 0.014).

**Table 5:** Agreement between expert malaria microscopy and the two algorithms on the 344 conclusive test results. Algorithm 1 is: first reading HRP2-line and subsequent confirmation with a *p*LDH-line; Algorithm 2 is: reading with a *p*LDH-line and confirmation with a HRP2-line.

	True positive	True negative	False positive	False negative
**Algorithm 1**	217 (63.1)	121 (35.2)	2 (0.6)	4 (1.2)
**Algorithm 2**	218 (63.4)	121 (35.2)	2 (0.6)	3 (0.9)

For the 344 “conclusive” diagnostic RDT results, the sensitivities, specificities, positive predictive values and negative predictive values were similar for algorithm 1 and 2 (Table 6). These values were 98.2% (217/221), 98.4% (121/123), 99.1% (217/219) and 96.8% (121/125) for algorithm 1, and for algorithm 2, 98.6% (218/221), 98.4% (121/123), 99.1% (218/220) and 97.6% (121/124).

**Table 6:** Diagnostic accuracy of algorithm 1 or 2 compared with expert microscopy (gold standard) for the diagnostic of *Plasmodium falciparum malaria* of 344 conclusive diagnostic test results obtained with sequential reading of two-line malaria RDTs.

	Sensitivity	Specificity	PPV	NPN
**Algorithm 1**	98.2 (95.4–99.5)	98.4 (94.3–99.8)	99.1 (96.5–99.8)	96.8 (92.0–98.8)
**Algorithm 2**	98.6 (96.1–99.7)	98.4 (94.3–99.8)	99.1 (96.5–99.8)	97.6 (92.9–99.2)

PPV = positive predictive value; NPV = negative predictive value.

When prior antimalarial treatment within the past 2 weeks was used to exclude malaria infection in children with inconclusive diagnostic result (hence considering them as being negative), the sensitivities, specificities, positive predictive values and negative predictive values calculated, were similar for algorithm 1 and 2. These values were 98.0%, 84.0%, 90.2% and 96.5% for algorithm 1 and 98.4%, 84.0%, 90.2% and 97.2% for algorithm 2, respectively.

Consequently, inadequate prescription of antimalarial observed with *Pf*HRP2-based RDT could be improved (26 cases when algorithms are used compared to 42 children when *Pf*HRP2-based RDT only is used). Moreover, malaria infection could be missed in 5 children when algorithms are used versus 4 children when *Pf*HRP2-based RDT is used [data not show].

## Discussion

The results of two diagnostic algorithms under evaluation demonstrated that the true and false results, which could not be diagnosed with single malaria RDT, could now be diagnosed and the test result can be classified in two groups: (i) the conclusive results for which malaria was diagnosed with a higher accuracy (>98%) following the algorithms; (ii) the inconclusive results for which malaria microscopy or additional investigation is required to better diagnose malaria in this group. The high sensitivity and specificity (>98%) of the algorithms for conclusive results only proofs that potential false positive and negative results could be identified by healthcare workers in a primary healthcare setting to improve the diagnosis of an ongoing infection. Consequently, healthcare workers are better able to accurately diagnose malaria in this group. This study also reported an association between the inconclusive results and the low malaria parasitemia, which is in line with other studies that reported that the low sensitivity of *p*LDH and the high sensitivity of *Pf*HRP2 leads to malaria false negative and positive results respectively. Moreover, after successful antimalarial treatments, *p*LDH antigen is metabolised within one week, but HRP2 antigens can persist in the blood up to 4 weeks and over. It is evident that low malaria parasitemia and persistence of HRP2 antigen after successful antimalarial treatment could lead to inconclusive diagnostic results (HRP2+/*p*LDH-) following the algorithms. A previous study reported that in a malaria endemic area, almost 50% of non-malaria infection due to bacteria and virus can be hide under a false positive HRP2 results [15]. Based on this observation, it is obvious that inconclusive diagnostic result reported in the present study is due either to low parasitemia or non-malaria infection occurred after a successful antimalarial treatment.

The association between false positive HRP2 results and previous antimalarial treatment within the past 4 weeks (or more) has been reported by Maltha et *al*. and Dalrymple et *al*. [4] [10]. It is therefore obvious that in absence of malaria microscopy, additional information on previous antimalarial treatment could be used to further establish the actual cause of fever in children with inconclusive test results. In the present study, information on previous antimalarial treatment partly resolved inconclusive diagnostic results, this is however a limited solution in the present study as only information on previous use in the last 2 weeks (and not 4 weeks) was collected. In routine practice, healthcare workers should firmly ask for information on previous malaria treatment within the past 4 weeks to further establish the actual cause of fever in children with inconclusive test results.

Despite the limit in the collection of information on previous antimalarial treatments to resolve the inconclusive results, the diagnostic accuracies for the algorithms were higher than either test band individually reported in the present study and those reported by Hawkes et *al*. (sensitivity = 88%; specificity = 82%) [12]. Consequently, the sequential interpretation of a two-line malaria RDT detecting both *Pf*HRP2 and *p*LDH could improve the diagnostic accuracy of malaria infection if the sequential interpretation proposed is supported by information on previous antimalarial treatment within the last 4 weeks in children with inconclusive results. In other words, the limitations of separately interpreting *Pf*HRP2 RDT, *p*LDH RDT or two-line malaria RDT detecting *Pf*HRP2 and *p*LDH can be greatly avoided. The sequential interpretation of the two-line malaria RDT detecting *Pf*HRP2/*p*LDH, supported by information on previous antimalarial treatment could improve the diagnostic accuracy. This approach can improve the management of febrile cases. The algorithms evaluated provided reliable alternatives to circumvent the diagnostic dilemma created by the use of a single malaria RDT. Malaria was accurately diagnosed with a diagnostic accuracy of 98.3% using diagnostic algorithm 1 or 98.6% with diagnostic algorithm 2. There were cases of febrile children (15.5%) in this study who had an “inconclusive” diagnostic test result and for whom expert malaria microscopy or other additional diagnostic and clinical investigations would be needed in order to do not miss potential treatable etiologies, including malaria. This indicates that both algorithm 1 and 2 can be helpful to accurately diagnose malaria in the majority of febrile children (84.5%). Both approaches eliminate doubts on the reliability of the diagnostic results achieved with the RDTs and the subsequent risk of having a malaria false positive or negative test result. The algorithms are designed for the diagnosis of *Plasmodium falciparum* only in area where *Pf*HRP2-based RDT are currently used. It is evident that the presence of non-*falciparum* species, such as *Plasmodium vivax*, could impact the interpretation of the algorithms.

The number of malaria diagnostics results that were considered “conclusive” in our study with either algorithm was higher (84.5%) than that reported by Murungi et *al* (65.9%) [18] who followed a similar approach. Although the more sensitive polymerase chain reaction testing was not performed in our study, the sensitivity and specificity of the malaria diagnostic testing reported by Murungi for children under– 5 years was almost similar to that reported in our study on conclusive diagnostic test results; i.e. 100% for Murungi versus 98% in our study. In our study, 4 malaria microscopy positive cases (2 *Plasmodium falciparum*, 2 other *Plasmodium* species) were missed by our approach. Furthermore, there were two cases reported positive by our RDT approach, but with expert microscopy no parasites were found in the blood slides.

A group of children (n = 63; 15.5%) remained undiagnosed as testing for malaria with the proposed algorithms did not result in a conclusive malaria diagnosis. These children were ill as they had fever and 23 of them had malaria infection confirmed by microscopy, prompting the need of additional testing if the RDT approach proposed in this study remains inconclusive. These cases were *Pf*HRP2+/*p*LDH- possibly due to previous anti-malarial treatment resulting in persisting HRP2 antigen [4] or the low sensitivity of *p*LDH which is likely not to detect often parasite level below 2,000 parasites/μl [19]. In absence of malaria microscopy, healthcare workers should pay more attention to these children because these inconclusive tests could conceal either a true malaria infection or non-malaria infections. In primary healthcare settings, nurses are responsible for health care and trained to manage uncomplicated infections based on clinical signs and symptoms, except malaria. So, it is evident that the information on the previous antimalarial treatment and non-malaria infections collected (based on signs and symptoms) could be helpful for clinical decision. Finally, clinicians should always pay particular attention to those children who are confirmed negative for malaria but do show clinical signs (like fever) as they may have other treatable causes of fever, such as bacterial infections [15].

This study was conducted in a seasonal malaria transmission setting and most samples were collected during the rainy season (July-October) when malaria transmission is high. Fewer children were included during the dry season (April-June). The intensity of malaria transmission could therefore influence the choice of which diagnostic algorithm is employed, but this has not been studied. Another potential limitation of algorithm 1 and 2 is in settings were HRP2 deletions occur [20] [21] [22]. In such a setting a HRP2-line will always be negative, even if an infection with *P*. *falciparum* is present. Consequently, in malaria endemic regions where HRP2 deletions are well established, the implementation of the proposed algorithms could increase the number of inconclusive results and misdiagnose some cases of malaria infection.

Finally, most likely the implementation of the proposed algorithms would be by sequentially using two different RDTs. However, this approach can double the time needed to confirm the diagnosis of a malaria infection as the result of the second test needs to be available too, and thismay also require taking a second blood sample. Research is needed towards patient and health care provider acceptability of such an approach.

## Conclusion

The strategy of sequential interpretation of two-line malaria RDT can improve the diagnosis of malaria by grouping the low malaria parasitemia and false HRP2 positive children in inconclusive test for which additional investigations on previous antimalarial treatments are required.
